# Exploring the role of soundscape in restorative experience: A pilot study from children’s perspective

**DOI:** 10.3389/fpsyg.2023.1131170

**Published:** 2023-03-14

**Authors:** Shan Shu

**Affiliations:** College of Architecture and Urban Planning, Qingdao University of Technology, Qingdao, Shandong, China

**Keywords:** children, classroom, restorative experience, soundscape, urban park

## Abstract

Indoor and outdoor noise is renowned for its ability to negatively affect children’s health and performance. However, the possible restorative benefits of everyday soundscapes in children are still poorly understood. This study aimed to explore the role of everyday soundscapes in children’s restorative experiences in frequented indoor (classroom) and outdoor (urban park) environments. In stage one, 335 children (7–12 years old) were interviewed using a questionnaire survey to investigate their restoration needs, restorative experience, and potential restorative sounds. In stage two, 61 children participated in a laboratory study to assess the perceived restorativeness of different soundscapes, which were combinations of potential restorative sounds and background noise, under signal-to-noise ratios (SNRs) from −5 to 15 dB. The findings denoted that the children’s need for restoration increased with age significantly. Younger children reported that the role of the sound environment was more important in their classroom experiences than in urban parks. Although the types of music displayed in surveyed parks were generally not preferred by the children, music was assessed as the most restorative sound in the laboratory study. Additionally, natural sounds were perceived to be more restorative than background noise in the context. In particular, birdsong showed more restorativeness in the classroom context, whereas fountain sounds showed more restorativeness in the park context. Additionally, an SNR of at least 5 dB is desirable when considering the restorative experiences of children in classrooms and urban parks.

## 1. Introduction

Extensive research has demonstrated that people’s perceptions of urban acoustic environments, including annoying noise and pleasant soundscapes, can significantly impact their health and wellbeing (Aletta et al., 2018). These influences might be more salient among children because their physical, mental, and cognitive health is at a high-speed development stage (Evans, 2006). Therefore, they are more sensitive and vulnerable to the surrounding environmental conditions, particularly acoustic environments, in comparison to adults (Mossberg, 2017). Over the last few decades, acoustic environments for children have attracted considerable attention. Several studies have noted the impact of environmental noise on children’s annoyance reactions, cognitive impairment, sleep disturbances, and cardiovascular responses (Evans et al., 2001; Stansfeld et al., 2005). However, despite increasing research evidence of acoustic importance, current acoustic environments in children’s everyday lives are still problematic. For instance, a questionnaire survey conducted in Netherlands with 1,311 school children revealed that noise was the most common annoyance, and most children (87%) complained that they were bothered by noise in their classrooms (Bluyssen et al., 2018). In a workshop with 335 children recruited to identify problems in their classroom by drawing or writing their choices on paper, the results showed that noise-related problems were more frequently reported than temperature, air, and light (Bluyssen et al., 2020). Besides, the influence of noise was much more serious on younger children. It is partly due to their less robust attentional abilities and less developed coping repertoire, which make younger children more easily distracted by auditory events. Another possible reason might be that younger children are noisier (Minelli et al., 2022). Overall, given the developmental vulnerability of childhood and the current precarious condition of the acoustic environment, it is essential and urgent to provide a healthy and supportive acoustic environment for children.

However, existing studies involving acoustic environments for children have mainly focused on the detrimental effects of annoying noise, especially traffic noise and noise generated by the children themselves, which are the most prevalent background sounds in their daily environments (Lercher et al., 2013; Estévez-Mauriz et al., 2020). To date, only a few have indicated the possible benefits of some noise on children’s performance (Söderlund et al., 2007), and even fewer have looked at the positive role of overall soundscapes in children’s experiences. However, there is a general consensus that a healthy acoustic environment cannot be achieved using only the conventional noise-mitigation approach (Ratcliffe, 2021). Moreover, neither traffic noise nor the noise generated by the children themselves can be ideally eliminated (Shield and Dockrell, 2004). Therefore, it is compelling to shift acoustic research from pathogenic noise to a salutogenic soundscape approach that concentrates on the promotion of health benefits, in addition to the mitigation of mental stress among children.

Recently, restorative environments have garnered interest in acoustic research because they are vital to address the public health problem induced by increased urbanization and stressful lifestyles (Hartig, 2007). As an important pathway in environmental perception, emerging studies have shown that natural soundscapes are commonly perceived as more restorative than urban soundscapes (Krzywicka and Byrka, 2017). Moreover, a growing amount of evidence suggests that natural soundscapes could effectively contribute to attention restoration and stress recovery based on objective measurements of cognitive performance and psychophysiological responses (Alvarsson et al., 2010; Medvedev et al., 2015; Zhang et al., 2017), although evidence remains limited since literature has emerged that offers contradictory findings on the actual restorative effect of natural sounds (Ma and Shu, 2018; Hedblom et al., 2019). Given the restorative benefits of soundscapes have been increasingly realized, more studies are being performed to investigate the mechanism of soundscape restorativeness in order to provide practical guidelines for soundscape design. For instance, the temporal-spectral composition of birdsong was indicated to play an important role in perceived restorativeness (Borker et al., 2020; Hong et al., 2021b). A questionnaire study conducted in five urban parks with 419 visitors showed audio-visual interaction and visitor characteristics could also significantly affect perceived soundscapes restorativeness (Guo et al., 2022).

It is important to note that the perceived restorativeness of soundscapes could be highly associated with their context because of the complicated interaction between sounds and human perception. A change in the visual context can significantly influence people’s responses to the acoustic environment. For example, Ma and Shu (2018) applied different audiovisual conditions to measure participants’ stress and fatigue recovery, and found a significant difference in the restorative effect of the same sound in different visual contexts. Likewise, an experiment conducted by Zhang and Kang (2022) indicated the strong effects of context on mood states in combination with urban sounds, and the effects varied across different sound types. Therefore, environmental contexts bear great weight in soundscape perception and their restorative effects on the population. However, existing studies of contexts in the soundscape field have mainly focused on the association of people and sound under a specific space and time. In particular, soundscapes in natural public spaces, such as rehabilitation gardens (Cerwén et al., 2016), urban parks (Zhang et al., 2019b), national parks (Wang et al., 2022), green spaces (Uebel et al., 2021), cemeteries (Nordh et al., 2017), and blue spaces (Liu et al., 2022), have received the most attention because they offer city dwellers a quiet environment to withdraw from urban noise and provide natural soundscape resources. As people spend most of their time indoors, the restorative benefits of natural sounds in indoor environments (e.g., offices, school classrooms, and hospitals) have received increasing research attention in recent years (Jahncke and Halin, 2012; Watts et al., 2016).

While the potential restorative benefits of soundscapes have been clearly outlined, research to date has tended to focus on adults rather than children. Considering that restorative experience varies across the life course, it might be thoughtless to apply adults’ restorative perceptions to children. Currently, school-aged children might experience high levels of mental stress and cognitive fatigue due to their competitive educational environment (Yang et al., 2015). Their current tendency toward alienation from the natural world is particularly concerning, which further aggravates their mental and physical health problems (Collado and Staats, 2016). Hence, there is an urgent need to establish restorative environments that might alleviate some of the negative symptoms of children’s contemporary lifestyles (Johnson et al., 2019).

Although limited in number, some studies have explored the restorative potential of children’s everyday life contexts, such as at home and in school classrooms (Korpela et al., 2002; Bagot, 2004). It is important to note that the framework of the restorative environment assumes a negative antecedent condition of either cognitive fatigue or psychological stress, making people feel depleted and in need of restoration (Kaplan, 1995). Previous studies typically assumed that children generally suffer from fatigue and stress during a typical school day (Shu and Ma, 2018), but the extent of children’s perceived need for restoration and their current restorative experience in their real life is still unclear, not to mention the possible difference between gender groups and developmental stages. Past studies have broadly shown a restorative advantage for natural environments as opposed to built environments (Dzhambov et al., 2021), but what is less certain are the specific attributes that make the environment restorative for children. Specifically, we assessed the dimension of sound appeal as contributing to restorative potential as children’s awareness and preference for environment sound strongly differ from those of adults (McAllister et al., 2019).

Recently, the restorative benefits of soundscapes for children have been demonstrated in a few studies. For example, an experiment of subjective evaluation conducted in a simulated classroom and urban park found that the restorative qualities of environmental sounds perceived by children were attractiveness, compatibility, and coherence (Shu and Ma, 2018). Moreover, empirical research has indicated that classroom soundscapes could provide significant restorative benefits to children’s reaction time and short-term memory (Shu and Ma, 2019), while urban park soundscapes can facilitate psychophysiological recovery among children to a certain extent (Shu and Ma, 2020). Nevertheless, research on the role of everyday soundscapes in children’s restorative experiences is insufficient. When introducing pleasant sounds into noisy environments to improve soundscape quality, both sound type and sound level have long been viewed as significant indicators of a desirable soundscape (Zhang et al., 2019a). However, existing literature has mainly focused on the impact of sound types, whereas few studies have examined the impact of sound levels. Additionally, whether children’s perceived restorativeness differs when applying restorative sounds in different visual contexts remains uncertain.

Given the above research gap, this study aimed to gain a holistic understanding of children’s restorative experiences and the possible benefits of everyday soundscapes in frequented environments. First, a questionnaire survey was conducted to explore the extent of children’s need for restoration and the sounds that might contribute to their restorative experience. A laboratory study was conducted to further examine the impact of sound type and level on children’s perceived restorativeness. This study is expected to expand the scope of restorative soundscape research and lay a theoretical foundation for the restorative design of children’s environments.

## 2. Methodology

This study included two sequential stages. In stage one, a questionnaire survey was conducted to explore children’s restoration needs, restorative experience, and everyday soundscapes with restorative potential in real classroom and urban park contexts. In stage two, a laboratory study was performed to further examine the children’s perceptions of typical potential restorative soundscapes in different contexts. Context variables except audio-visual factors were ruled out, as soundscapes may not reach their full potential for restorative benefits when children were distracted by other things while they were in real settings (e.g., companions, mobile phones) (Zijlema et al., 2017).

The research was conducted in accordance with the Declaration of Helsinki. Ethical approval was obtained from the Academic Committee of the School of Architecture at Tianjin University. All the children and their parents (or accompanying guardians) were informed about the study protocol, and they freely volunteered to participate in the study. Oral consent from children and written informed consent from their parents (or accompanying guardians) were obtained before the study.

### 2.1. Stage one: Questionnaire survey

#### 2.1.1. Survey sites

Considering the environments frequented by children, school classrooms and urban parks were selected as sites for the questionnaire survey. School classrooms are typical indoor environments that are commonly identified as cognitive-demanding contexts, whereas urban parks are outdoor environments that were identified as mental-relaxing contexts. As familiar contexts for children, the restorative potential of soundscapes in these places might have an indispensable role in children’s environmental experiences.

**FIGURE 1:**
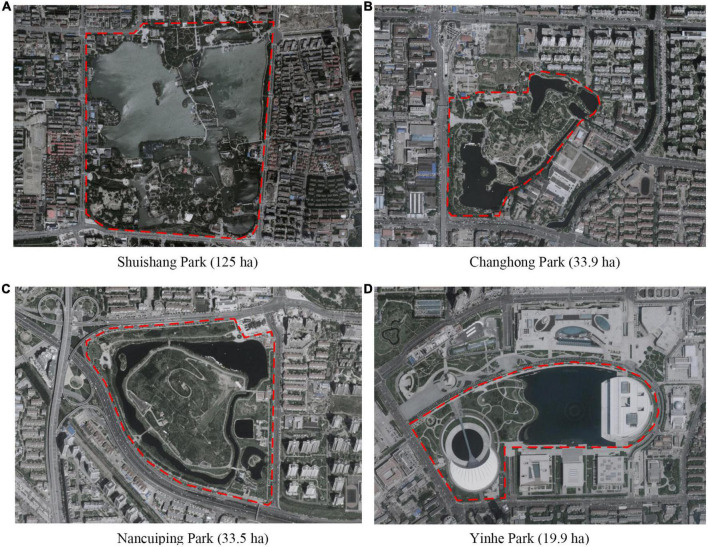
Satellite views of four survey urban parks. **(A)** Shuishang Park; **(B)** Changhong Park; **(C)** Nancuiping Park; **(D)** Yinhe Park.

For the selection of school classrooms, primary school children from grades one to six were interviewed voluntarily, including school children of all ages. The selected classrooms ranged from 50 m^2^ to 60 m^2^ in size and were occupied by approximately 40 children, which is typical in Chinese primary schools. For the selection of urban parks, four parks in Tianjin were selected to represent urban parks of different sizes and features (Figure 1): Shuishang Park (a municipal-level comprehensive park, 125 ha in area), Changhong Park (a district-level comprehensive park, 33.9 ha in area), Nancuiping Park (a natural urban park, 33.5 ha in area), and Yinhe Park (a cultural urban park, 19.9 ha in area).

#### 2.1.2. Respondents

During the questionnaire survey, children aged seven to 12 years were selected as respondents. In total, 353 questionnaires were randomly distributed, and 335 were completed in full and used for analysis. The demographic information of all the respondents is presented in Table 1. A total of 188 children in six school classrooms and 147 children in four urban parks were interviewed. The sample size for each age and gender was comparable: 170 boys and 165 girls (mean age = 9.3 years, SD = 1.72). All children reported normal hearing and normal or corrected-to-normal vision.

**TABLE 1:** Demographic information of children interviewed in classrooms and urban parks.

Group		Classrooms		Urban parks		Total	
		*N*	%	*N*	%	*N*	%
Gender	Boys	99	29.6	71	21.2	170	50.7
	Girls	89	26.6	76	22.7	165	49.3
Age	7	41	12.2	23	6.9	64	19.1
	8	26	7.8	34	10.1	60	17.9
	9	36	10.7	22	6.6	58	17.3
	10	29	8.7	30	9.0	59	17.6
	11	32	9.6	17	5.1	49	14.6
	12	24	7.2	21	6.3	45	13.4
Total		188	56.1	147	43.9	335	100

#### 2.1.3. Questionnaire

The questionnaire for children consisted of four sections: (1) children’s restoration needs in their everyday life, including the need for “stress reduction” and “fatigue recovery,” which are the two dominant indicators of a restorative environment (Kaplan, 1995). The restoration need was assessed by a five-point scale ranging from “not at all” to “extremely”; (2) the potential restorative factors of the place, which were collected *via* open-ended questions asking about the environmental factors that could make children feel relaxed and pleased. A list of environmental factors was presented as examples to make the question more intelligible for children; (3) the importance of the acoustic environment in their restorative experience of the survey site, assessed by a five-point scale ranging from “not at all” to “extremely”; (4) the potential restorative sounds for children, including the sounds they were familiar with or preferred. Notably, familiarity and preference were commonly associated with perceived restorativeness as indicated in previous research (Korpela et al., 2002; Staats et al., 2003). Potential restorative sounds were collected *via* open-ended questions, supplemented by a list of sounds presented in the questionnaire. To design a qualified questionnaire for the target children group, the wording of each question was adapted to match children’s vocabulary and experiences through interviews with some young children. Additionally, before administering the formal survey, the questionnaire was piloted among 24 children of different ages to ensure it could be finished in a reasonable amount of time (around 10 min) and could be comprehended easily and accurately by the target children group.

### 2.2. Stage two: Laboratory study

#### 2.2.1. Participants

In the experiment, 61 children (mean age = 10.21 years, SD = 1.18), including 27 boys and 34 girls, were recruited *via* social media and snowball sampling from various primary schools in Tianjin, China. All children reported normal hearing and normal or corrected-to-normal vision.

#### 2.2.2. Environmental contexts

**FIGURE 2:**
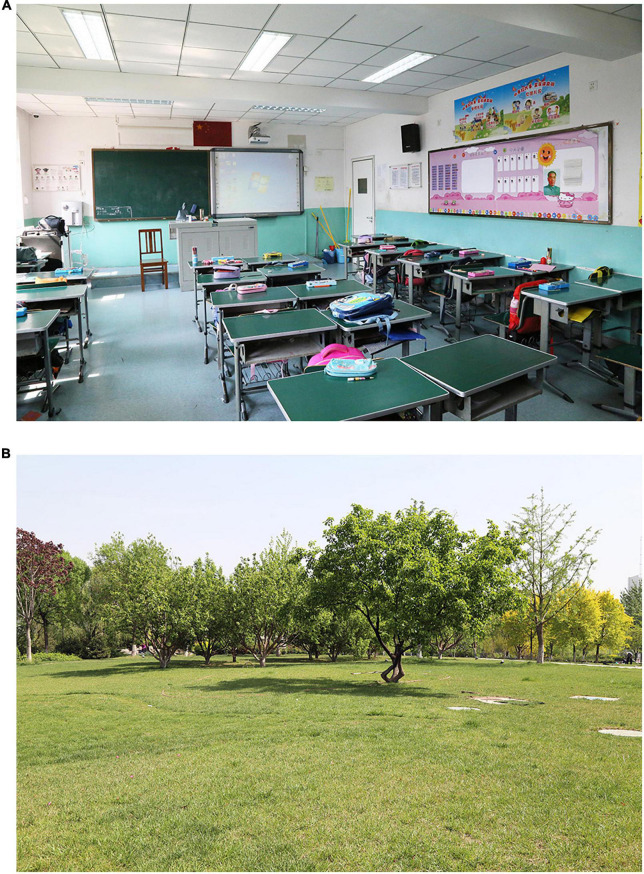
The visual contexts of a classroom **(A)** and an urban park **(B)**.

Consistent with the questionnaire survey, this study was also conducted in two simulated contexts: school classrooms and urban parks. To select the representative pictures utilized in the study, many photos of classrooms and urban parks were first captured during the questionnaire survey. Then, a preliminary test was conducted with the children to select the photos of the two contexts in terms of representativeness. A detailed explanation of photograph selection can be found in our previous study (Shu and Ma, 2018). Figures 2A, B shows photos that were finally chosen as visual contexts. It is important to note that the presence of people was avoided in the pictures, as it was regarded as a significant factor influencing the restorative experience (Wang et al., 2016). During the experiment, the photos were shown on a large 46-inch screen placed in front of the participants at a distance of approximately 100 cm.

#### 2.2.3. Sound stimuli

**FIGURE 3:**
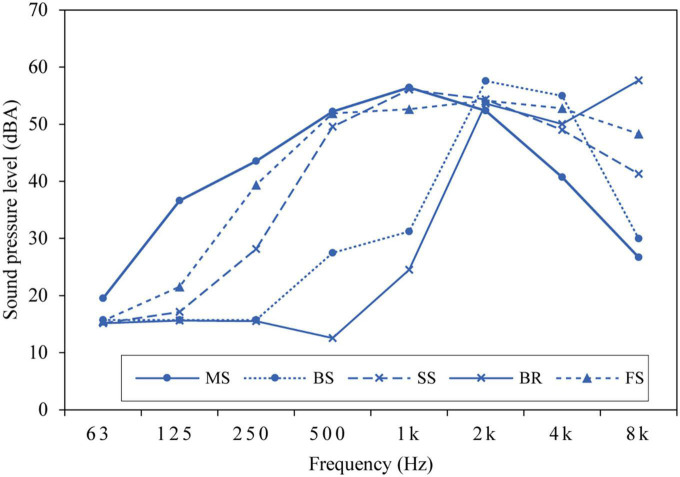
The spectra of the five potential restorative sounds. MS, music; BS, birdsong; SS, stream sound; BR, bell ring; FS, fountain sound.

This stage of research was designed to further examine children’s restorative perceptions of individual soundscapes in a laboratory setting, under controlled conditions. The selection of sound stimuli was based on the following criteria: (1) they were generally preferred by children in stage one; (2) they would be easy to add to children’s living environment through corresponding soundscape design, such as green-blue space planning and landscape accessories designing. Thus, five restorative sounds (music, birdsong, stream sound, bell ring, and fountain sound) were selected for the trials. The music was “Souvenirs d’enfance,” a piece of calm and relaxing piano music without lyrics that adhered to the criteria of clarity and high quality. Birdsong is referred to as chirps of sparrows and some other bird species that are common in parks. Stream sound was generated by a stream waterscape with slightly tilted stone steps. The bell ring was produced by irregularly striking a wind chime with suspended metal bells. The fountain sound came from a jet-and-basin fountain in the park. The spectra of the five restorative sounds are presented in Figure 3. It can be seen that the music was dominated by mid-frequency components, while the birdsong and bell ring predominantly contains high-frequency components above 2.0 kHz. The stream sound and fountain sound is very broadband with middle-high frequencies.

In addition, background noise was used as a reference for acoustic comparison, which was different in the two contexts. The background noise of the classroom was recorded in an unoccupied classroom during recess time to match the visual context in Figure 2A, and it mainly included external noise generated by the children and equipment in the building. The noise of urban parks was recorded in a typical urban park in Tianjin on a normal weekend day, including talking, activity sounds, music, and murmurs of distant urban traffic.

All the sound recordings were collected using a portable digital recorder (Sony PCM-D50, Japan, sampling frequency: 44.1 kHz, resolution: 16 bit) comprising two external microphones in a 90°XY configuration with windshield, and it is able to collect sound signals over dual channels. To avoid the influence of environmental noise, we conducted recordings of restorative sounds in relatively quiet areas without other noticeable prominent sounds from people talking, road traffic, etc. The sound recordings were at least 10 min each, with 30-s of steady performance in the middle of the recordings excerpted and used in the experiment.

In addition to sound type, this study was also conducted to explore children’s restorative perceptions of different sound pressure levels in contexts with certain background noise, which is inevitable for soundscape experience in real-life contexts. Therefore, the indicator of signal-to-noise ratio (SNR) was introduced in this study to design the acoustic stimuli (Hong et al., 2021a), which were combined with potential restorative sounds (signal) and background noises (noise). While the background noise levels were pinpointed according to the current noise standard and kept constant, the potential restorative sound levels varied with SNRs. Specifically, the noise level in the classroom was set at 45 dBA according to the upper limit of the noise standard set for primary schools in China, and the noise level in the urban park was set at 55 dBA according to the environmental quality standard for noise in Tianjin. Each of the combined acoustic stimuli was given under five different SNR levels, ranging from −5 dB to 15 dB in intervals of 5 dB. Correspondingly, the a-weighted equivalent sound pressure level of the five restorative sounds was 40–60 dB (A) in the classroom and 50–70 dB (A) in the urban park. Altogether, 26 acoustic stimuli (five restorative sounds × five SNRs + one background noise) were yielded in the context of the classroom and urban park, respectively (Table 2).

**TABLE 2:** The acoustic stimuli in the experiment.

Context	Sound type	Restorative sound dB (A)	Background noise dB (A)	Total dB (A)	SNR dB
Classroom	Music	40.0	45.0	46.3	−5 dB
	Birdsong	45.0		48.1	0 dB
	Stream sound	50.0		51.2	5 dB
	Bell ring	55.0		55.6	10 dB
	Fountain sound	60.0		60.1	15 dB
	Background noise	–		45.0	–
Urban park	Music	50.0	55.0	56.2	−5 dB
	Birdsong	55.0		57.9	0 dB
	Stream sound	60.0		61.0	5 dB
	Bell ring	65.0		65.6	10 dB
	Fountain sound	70.0		70.2	15 dB
	Background noise	–		55.0	–

The editing, calibration, and mixing of the acoustic stimuli was processed using Adobe Audition software. First, the background noises of the recordings were reduced in the software. Second, given that the sound stimuli were reproduced through a headphone during the experiment, the sound level of each recording was calibrated using a Norsonic Nor140 (Norway) Class 1 sound level meter, which was placed near the headphone (AKG K702) driven by a soundcard (YAMAHA Steinberg UR242, China), to ensure the accuracy of the emitted signal with respect to the real sound. Then, each restorative sound was mixed with the background noise to produce the final acoustic stimuli with different SNRs. The same headphone and soundcard setup was used to present the sound stimuli to the participants. Notably, the duration of each sound material was set at 2 min because, according to the preliminary experiment, children generally began to show impatience at 2 min and this duration was adequate for children to complete the questionnaire.

#### 2.2.4. Measurement

The perceived restorative sound scale for children (PRSS-C) was used to assess the children’s restorative perceptions of soundscapes. The PRSS-C was designed according to the demographic characteristics of primary school children, and it was shown to successfully differentiate the restorativeness of environmental sounds assessed by children in our previous study (Shu and Ma, 2018). The scale consists of 16 items (e.g., I found this sound appealing; this sound environment fits my personal preference; the sound I am hearing belongs here). Each item was assessed on a five-point scale in response to “How much do you agree with the statement…?” (0 = not at all, 1 = slightly, 2 = moderately, 3 = very, 4 = extremely). Children’s personal information, such as age and gender, was also collected. The Cronbach’s alpha of the PRSS-C was over 0.80, indicating the internal consistency of the questions.

#### 2.2.5. Procedure

The experiment was performed in a semi-anechoic chamber at Tianjin University, which is commonly used as an ideal listening room to avoid disturbance from outside sound and to ensure that the sound environment is kept constant with low background noise (20–25 dBA). Children were accompanied by their parents who waited in a space outside the chamber during the experiment.

In this study, restorative soundscapes were assessed with a two-contexts (classroom, urban park) between-subjects design. The children were randomly assigned to one of the contexts (classroom or urban parks). Overall, 30 children participated in the experiment in the context of a classroom and 31 children in the context of an urban park. The gender and age of the two groups were kept comparable to minimize group differences. In each context, the children were presented and asked to evaluate 26 acoustic stimuli (five restorative sounds, five SNRs, and one background noise). During the experiment, the children were first asked to imagine a scenario that induced attention fatigue and stress (Staats et al., 2003; Shu and Ma, 2018; Ratcliffe et al., 2020). Since the experiment was conducted during the summer holiday when children had just finished their final exams, the scenario has been revised according to children’s actual conditions, and it was pre-tested among 10 children based on conceivable and familiar. Researchers read the text as follows: “This semester, you have studied very hard. Now, at the end of the final exams, you have been asked to do a lot of homework. You have difficulty concentrating, and you are very anxious because of concerns about test scores.” Then the audio-visual stimuli were presented, while the children were asked to answer the questionnaire. There was a 10 s interval to refresh before exposure to the next audio-visual stimuli, and it approximately took 45 min on average to complete the experiment.

### 2.3. Data analysis

Statistical analyses were performed using IBM SPSS Statistics 25.0. As for the questionnaire data collected in stage one, the ratings on the ordinal scales (restoration need and acoustic environment importance) failed to meet normality assumptions, as assessed by the Kolmogorov–Smirnov normal test. As such, non-parametric tests were used to examine the influence of gender and age on these ratings. Children’s responses to open-ended questions (restorative factors and sounds) were calculated in percentage because the frequency with which a sound source was mentioned can provide the first clue about potential restorative factors and sounds.

As for the data collected in stage two, the mean values of each PRSS-C were firstly calculated. As the assumption of normality was also not satisfied, Kruskal–Wallis tests were applied to identify the influential factors for PRSS-C values within each context, with the independent variables being sound type and SNR. *Post-hoc* pairwise comparisons with Bonferroni adjustments were performed to test the differences between the sound types and SNRs. Additionally, Mann–Whitney U tests were conducted to compare the differences between the context of the classroom and urban park across all sound types and SNRs. We also tested the effects of age and gender on perceived restorativeness. The present study considered *p*-values of < 5% as statistically significant.

## 3. Results

### 3.1. Stage one: Children’s restoration need, experience, and potential restorative sounds

**FIGURE 4:**
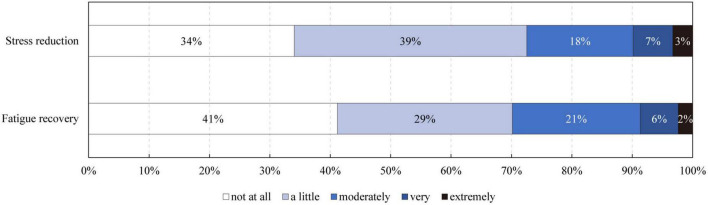
The percentage of children that need stress reduction and fatigue recovery.

The percentage of children needing to restore from stress and fatigue was counted (Figure 4), and the outcomes showed that more than half of the children reported at least “a little” need for both stress reduction and fatigue recovery, indicating that they generally need restoration in their daily lives. Overall, 66.0% of children reported the need for stress reduction, and 58.8% of children reported the need for fatigue recovery. Notably, around 10% of children reported they were “very stressful/fatigued” or “extremely stressful/fatigued.”

**FIGURE 5:**
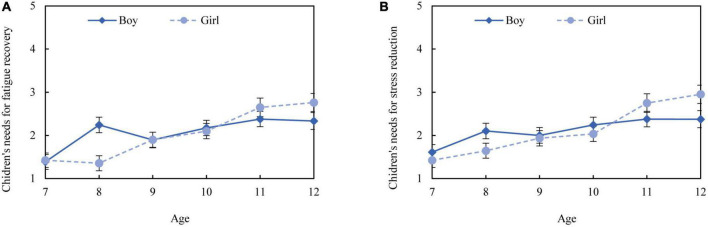
Difference in restoration needs for fatigue recovery **(A)** and stress reduction **(B)** among gender and age.

The non-parametric tests were conducted to examine the influence of age and gender on children’s need for restoration. The results showed a significant difference in both stress reduction and fatigue recovery between age groups (*p* < 0.001), but there was no substantial difference between the genders (*p* > 0.05). As shown in Figure 5, children’s need for stress reduction and fatigue recovery gradually increased with age. Moreover, boys’ need for restoration was higher than girls’ before 9 years of age, while girls reported more restoration needs than boys after 10 years of age.

**FIGURE 6:**
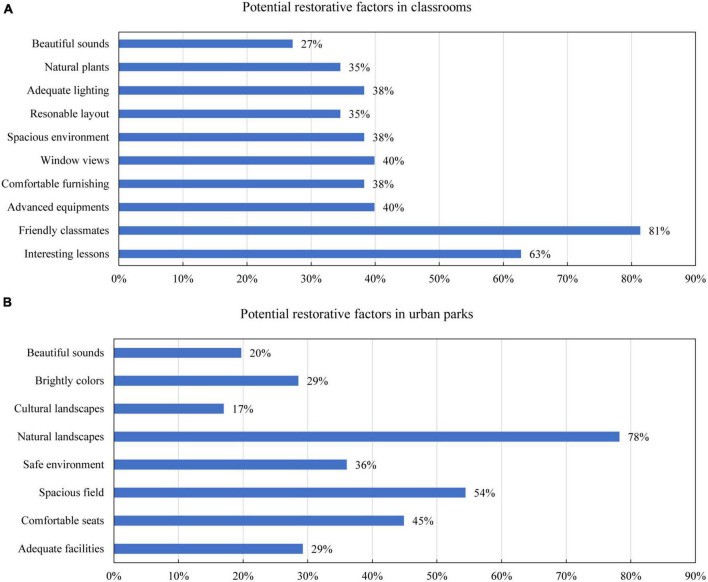
The potential restorative factors in classrooms **(A)** and urban parks **(B)**.

The results of the questionnaire survey also showed large differences in restorative environmental factors between classrooms and urban parks. Figure 6A highlights that the most restorative environmental factors were “friendly classmates” and “interesting lessons” in classrooms, which were reported by 81 and 63% of the children, respectively. Other environmental factors, such as equipment, sound, light, layout, space, window scenery, and furniture, have also been reported to have restorative potential in > 30% of children. Conversely, Figure 6B signifies that the most restorative environmental factors in urban parks were “natural landscape” and “open field,” which were reported by 78 and 54% of the interviewed children, respectively. Meanwhile, “comfortable seats” and “safe environment” were mentioned by 45 and 36% of the children.

**FIGURE 7:**
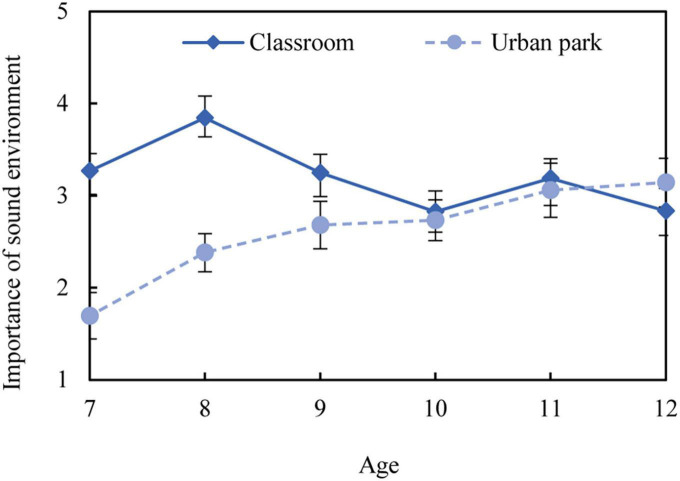
The importance of a sound environment in children’s restorative experience.

The importance of sound environments in classrooms and urban parks was explored through the questionnaire survey. In total, 89.9% of the children in school classrooms and 82.3% of the children in urban parks reported that the sound environment was at least “a little” important to their environmental perceptions. Additionally, 10 and 28% of the children reported the classroom sound environment was “very important” and “extremely important,” respectively. However, only 7 and 8% of the children reported urban park sound environment was “very important” and “extremely important,” respectively (Figure 7). Moreover, a Mann–Whitney U test showed that the sound environment in classrooms was much more important than that in urban parks, as assessed by children (*p* < 0.001).

**FIGURE 8:**
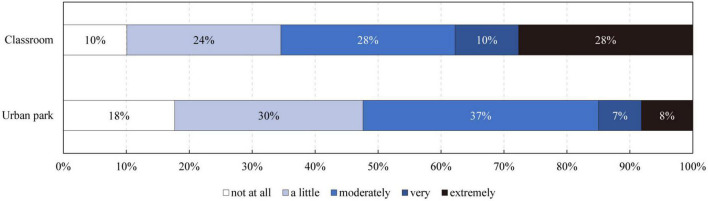
Difference in sound environment importance.

The non-parametric tests were conducted to examine the differences in sound importance assessment between gender and age. The findings outlined a significant difference among ages in urban parks (*p* < 0.001) as well as a slight difference in classrooms (*p* = 0.088). Figure 8 shows that for children aged between 10 and 12 years, the sound environment in classrooms was as important as in urban parks. However, for children younger than 10 years old, the acoustic environment proved to be much more important in the classroom. No significant difference was found between the genders in either context (*p* > 0.05).

**FIGURE 9:**
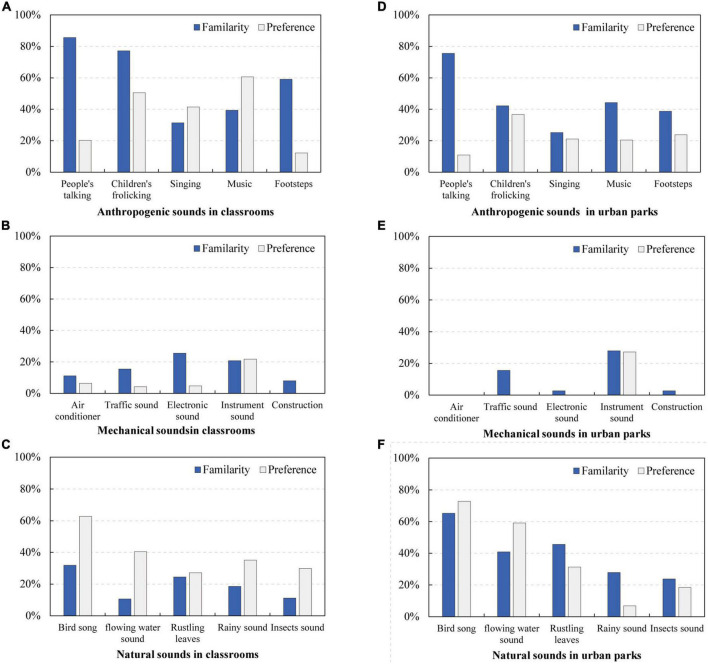
Potential restorative sounds reported by children. **(A)** Anthropogenic sounds in classrooms; **(B)** mechanical sounds in classrooms; **(C)** natural sounds in classrooms; **(D)** anthropogenic sounds in urban parks; **(E)** mechanical sounds in urban parks; **(F)** natural sounds in urban parks.

The percentage of each potential restorative sound (assessed by familiarity and preference) is presented in Figure 9.

In the classroom setting, the most familiar sound sources for children were anthropogenic sounds, such as people’s talking, children’s frolicking, and footsteps, as reported by more than half of the children (refer to Figure 9A). Contrarily, natural sounds and mechanical sounds were much less familiar, reported by less than 30% of the children (refer to Figures 9B–C). For preference, birdsong was preferred by 63% of children, followed by the sound of music and children’s frolicking, which were chosen by 61 and 51% of children, respectively (refer to Figures 9A, C). Altogether, natural and anthropogenic sounds were preferred in classrooms, while mechanical sounds were the least familiar and preferred by children.

In the setting of urban parks, the most familiar sound sources for children were anthropogenic sounds (i.e., talking, music, and children’s frolicking) and natural sounds (i.e., birdsong, rustling leaves, and flowing water). However, natural sounds seemed to be preferred by children over anthropogenic sounds. For example, birdsong and water sounds were preferred by 73 and 59% of children, respectively (refer to Figure 9F). However, all anthropogenic sounds were preferred by less than 40% of children (refer to Figure 9D). Additionally, mechanical sounds were the least familiar and preferred sounds for children in urban parks, and this result was similar to that of classrooms (refer to Figure 9E).

### 3.2. Stage two: Perceived restorativeness of different soundscapes

**FIGURE 10:**
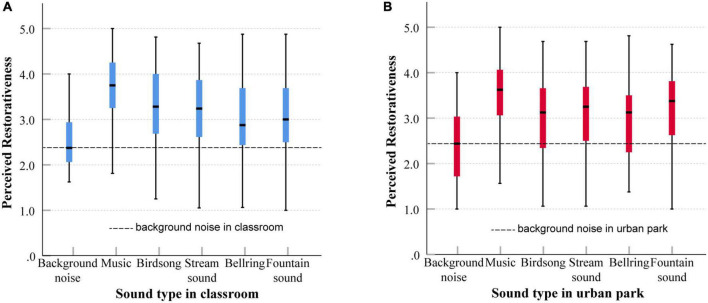
Boxplots of perceived restorativeness of each sound type in the context of a classroom **(A)** and urban park **(B)**, showing median, interquartile range, maximum, and minimum values.

Children’s restorative perceptions of different soundscapes were explored in a laboratory setting. Nonparametric analysis was performed to investigate the influence of different sound types and SNRs on children’s restorative perception. For sound types, the Kruskal–Wallis test yielded a significant difference in overall perceived restorativeness within the classroom (χ^2^[5] 86.14, *p* < 0.001) and within the urban park (χ^2^[5] = 63.96, *p* < 0.001). Boxplots of the perceived restorativeness of each sound type are presented in Figure 10. The pairwise comparisons showed that the perceived restorative scores of music, birdsong, stream sound, bell ring, and fountain sound were all significantly higher than the scores of background noise, while music was rated as significantly more restorative than other sound types, and the results were the same in both contexts. In the context of the classroom, birdsong was rated as significantly more restorative than bell ring (*p* = 0.012) and fountain sound (*p* = 0.004). However, in the context of urban parks, the fountain sound was rated as significantly more restorative than birdsong (*p* = 0.045) and bell ring (*p* = 0.002).

Table 3 compares the perceived restorativeness of each sound type between the classroom and urban park contexts. The results showed that the perceived restorativeness of the birdsong and fountain sounds differed significantly between the two contexts. Specifically, birdsong was perceived to be much more restorative in classrooms (*p* = 0.010), while fountain sounds were perceived to be much more restorative in urban parks (*p* = 0.020). No significant differences were found in the perception of other sound types between the two contexts.

**TABLE 3:** Comparison of perceived restorativeness of each sound type between a classroom and urban park.

Sound type	Classroom		Urban park		Z (w)	*p*
	Median	IQR	Median	IQR		
Background noise	2.38	0.91	2.44	1.44	−0.05	0.629
Music	3.75	1.00	3.63	1.00	−1.72	0.085
Birdsong	3.28	1.33	3.13	1.38	−2.56	**0.010**
Stream sound	3.19	1.27	3.25	1.19	−0.18	0.858
Bell ring	2.88	1.27	3.13	1.25	−1.07	0.285
Fountain sound	3.00	1.19	3.38	1.19	2.32	**0.020**

Bold values mean *p* < 0.05.

**FIGURE 11:**
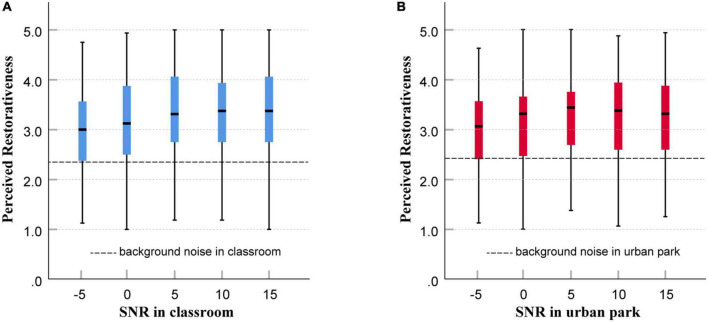
Boxplots of perceived restorativeness of each signal-to-noise ratio (SNR) in the context of a classroom **(A)** and urban park **(B)**, showing median, interquartile range, maximum, and minimum values.

The influence of SNRs was also analyzed using the Kruskal–Wallis test. A significant difference in restorativeness was found among SNRs in the classroom (χ^2^[4] 20.87, *p* < 0.001) and in the urban park (χ^2^[4] 13.23, *p* < 0.001). *Post-hoc* pairwise comparisons showed that the perceived restorativeness was significantly higher when the SNR was 5–15 dB compared to an SNR of −5 dB in both contexts. Boxplots of the perceived restorativeness of each SNR are presented in Figure 11. Children’s overall perceived restorativeness increased with the SNRs and reached a maximum when the SNR was 5 dB. However, a tendency to decrease was witnessed when the SNR exceeded 5 dB in the context of urban parks.

Table 4 compares the perceived restorativeness of each SNR between the classroom and urban park contexts. The results showed no significant difference in restorative perceptions of SNRs between the two contexts.

**TABLE 4:** Comparison of perceived restorativeness of each SNR between a classroom and urban park.

SNR	Classroom		Urban park		Z (w)	*p*
	Median	IQR	Median	IQR		
−5	3.00	1.20	3.06	1.19	−0.04	0.969
0	3.13	1.39	3.31	1.25	−0.16	0.875
5	3.31	1.31	3.44	1.06	−1.11	0.267
10	3.38	1.19	3.38	1.38	−0.71	0.479
15	3.38	1.31	3.31	1.31	−0.83	0.407

Additionally, the nonparametric Spearman correlation test indicated a significant positive relationship between age and children’s perceived restorativeness only in the classroom context (*r* = 0.123, *p* < 0.001). Moreover, a significant gender difference was found in the context of the urban park according to the Mann–Whitney U-test (χ^2^ 0.123, *p* < 0.001), which showed that the soundscapes were perceived to be more restorative for girls than for boys.

## 4. Discussion

In this study, a questionnaire survey and laboratory experiment were successively conducted to explore the role of everyday soundscapes in children’s restorative experiences.

In stage one, the questionnaire survey demonstrated that children aged seven to 12 generally have a need for restoration. Nevertheless, it is essential to note that the need for restorative environments increased with age, and the increasing need was more salient in girls. The higher restoration needs of older children may be explained by the fact that they suffer from environmental stress for a longer period of time and their school tasks require higher mental and cognitive consumption. The difference between genders was in line with previous research, which specifies that girls are more sensitive to environmental risks as girls complained more about noise, temperature, and air inside the classroom than boys (Bluyssen et al., 2020). These findings suggest that it is imperative to explore restorative environments for older children, particularly girls. This study also demonstrated that the surveyed classrooms and urban parks generally showed positive restorative potential for children, and their restorative experiences in classrooms and urban parks were highly related to their functional and spatial properties, respectively. Therefore, different environmental properties should be considered in classrooms and urban parks to promote children’s restorativeness. Specifically, promoting interesting education and a friendly environment is key to a restorative classroom, whereas creating open and natural spaces is a priority in restorative parks.

Additionally, a sound environment is generally reported to be a crucial element in children’s restorative experiences. It is interesting to note that children younger than 9 years old reported that the sound environment in classrooms was much more important than that in urban parks, while older children reported that they were equally important. This finding is consistent with previous research, which suggests that younger children are more annoyed than older children while learning in a noisy classroom (Klatte et al., 2013). However, the sound environment in urban parks is not as important as expected for younger children. Younger children appear to be unaware of their surrounding sound environment, and they have a tendency to perceive sounds in a somatic rather than a psychological manner, as indicated by previous studies (Waye et al., 2013). Therefore, the positive benefits of park soundscapes might manifest mainly in children’s bodily reactions instead of mental reactions. Nevertheless, this finding confirms the importance of age differences in acoustic research for primary-school children.

Regarding potential restorative soundscapes, assessed by both familiarity and preference, music was mentioned by most of the interviewed children in classrooms, followed by children’s frolicking. This result differs somewhat from earlier studies with adults, which identified noise generated by children as a predictor of decreased preference and restorative value (Deng et al., 2020). This discrepancy is likely related to the social meaning of restorative environments. While adults prefer to be alone in restorative environments to avoid unnecessary social interaction with others, for children, engaging in cooperative activities with the company of friends appears to be a key factor leading them to perceive a setting as restorative (Hartig, 2004). The restorative potential can also be associated with audible safety (Andringa and Lanser, 2013; van den Bosch et al., 2018). For children, the presence of frolicking is a kind of audible indication of safety, which allows the freedom of mind to engage in proactive behavior for quality of life and health optimization; therefore, it was identified as a restorative soundscape. It is also important to note that birdsong and stream sounds were highly preferred by children (> 40%), although they were not frequently heard in school classrooms, which indicates these sounds could offer considerable restorative potential for children *via* appropriate schoolyard soundscape design. In urban parks, natural sounds (i.e., birdsong and flowing water sounds) were the most restorative sounds, which is consistent with previous studies that have verified the restorative effects of natural soundscapes on adults. Unlike classroom findings, music was much less mentioned by children as a preferred sound in urban parks. This is mainly because the music in classrooms was generally light or pop music played back during music class or recess time, whereas singing from older people or square-dancing music that they generally disliked was widely reported during the survey in urban parks. Therefore, appropriate music based on children’s preferences could be considered in urban parks to provide children with a restorative environment. On the other hand, children’s preference for music might indicate that the classroom soundscape is suboptimal. Since music is an individual mood regulator, the compatibility of music and individual needs is particularly important, which should be carefully considered in classrooms with different children groups (Kaplan, 1995).

In stage two, the laboratory experiment results confirmed that the sound of music, birdsong, stream sound, bell ring, and fountain sound could substantially improve the perceived restorativeness of soundscapes in the classroom and urban park by combining them with background noise. Additionally, a significant difference was identified across these soundscapes. Among them, music was the most restorative soundscape in both contexts, which confirms the restorative benefits of music based on children’s subjective perceptions (Shu and Ma, 2018, 2020) and indicates that the restorativeness of music may be independent of context. In other words, children’s restorative perception of music does not vary across different environmental contexts, and it might be dominated by the characteristics of the music itself, given that music in urban parks was not preferred by many children during the questionnaire survey. Consistent with the results from the questionnaire survey, birdsong was also perceived to be highly restorative in the laboratory setting. Notably, compared to birdsong in the context of an urban park, it was perceived to be significantly more restorative in the classroom. It might be because birdsong is part of a restorative whole in the park, while it is acoustically unique in the classroom. Therefore, it is more fascinating and promotes feelings of “being away.” This result indicates that the restorative potential of birdsong in classrooms is far from being explored through appropriate landscape designs in primary schools. In contrast to birdsong, fountain sounds in the classroom were much less restorative than those in the urban park, and the findings of the field survey and laboratory experiment were generally consistent. It might be mainly due to the broadband masking effects of the fountain sound on speech, which makes the classroom less ideal for communication. The difference in fountain sounds between the two contexts might also due to the congruence of the sound with its visual scenes. As reported by some children after the experiment, fountain sounds were frequently heard in urban parks. Contrarily, it is rarely heard and seems incompatible in classrooms, making children feel antipathetic rather than restorative. In all, the sounds that were preferred in field settings were generally perceived to be restorative in the laboratory setting. More importantly, some sounds (e.g., music) are context-independent, whereas other sounds (e.g., birdsong and fountain sounds) should be considered depending on their visual contexts.

Additionally, SNR was found to strongly influence children’s restorative perception of soundscapes with a certain background noise level, which complied with the national standards exactly (i.e., 45 dBA in classrooms and 55 dBA in urban parks). Children assessed the soundscapes to be most restorative when the SNRs reached 5 dB; however, there was a trend of decrease when the SNRs reached 15 dB. Thus, 5 dB might be the most efficient SNR when considering the restoration of soundscapes for children. Nonetheless, the restorative SNRs should be considered with caution if they are applied in real-life environments, given that the restorative SNRs might be higher in actual environments than in laboratory environments as other environmental factors (e.g., vision, temperature, humidity, etc.) *in situ* environments might have reduced the participants’ attention to acoustic stimuli (Hong et al., 2021a). In addition, it should bear in mind that SNR is not the optimal indicator to explain children’s restorative perception of the whole soundscape, and perceived audibility of the sound sources is the key point. Audibility denotes the capacity of sound to be perceived by people, and it is a comprehensive sensation influenced by the hearing ability of the listener, the masking effects of other sound sources, and by the frequency content and amplitude of the sound. In this way, there is a pathway from acoustic characteristics (e.g., SNR) to audibility and ultimately, the restorative quality, which should be further explored to better explicitly explain the cognitive mechanism between acoustics and restoratives.

Despite the findings of this study, a few limitations must be addressed in future research. First, the selection of survey sites, especially school classrooms, was voluntary, which could introduce a potential bias in the results. Therefore, the findings may not be generalizable to the entire primary school population. Second, in addition to the quantitative data obtained from our study, interviews, observations, and other qualitative measures could be considered in future studies to provide a more comprehensive understanding of how the process of restoration works for children. Despite these limitations, the results of this study provide a more holistic understanding of restorative soundscapes from a children’s viewpoint.

## 5. Conclusion

Based on the results of the in-situ questionnaire survey and laboratory experiments, the following conclusions were drawn:

(1) Children’s need for restoration increased with age, and the current restorative experience of classrooms and urban parks mainly depended on their functional and spatial properties, respectively.

(2) The role of the sound environment is more important in the restorative experience of classrooms than in urban parks for young children. Among all the environmental sounds, natural sounds showed the most restorative potential *in situ*.

(3) In laboratory settings, however, music was perceived to be the most restorative sound, followed by natural sounds. In particular, the restorativeness of birdsong and fountains is context-dependent.

(4) Signal-to-noise ratio (SNR), age, and gender can significantly influence children’s restorative perceptions of soundscapes. Soundscapes seem to facilitate more restorative benefits for older children and girls.

This study offers theoretical contributions to restorative soundscapes from the perspective of children. It is among the first to systematically investigate whether, what, and how soundscapes interfere with children’s restorative experience. Additionally, the results of this study have generated implications for both soundscape and urban design aimed at children. In particular, sound type, sound level and visual context consistency were important metrics when designing restorative soundscapes for children. Children’s age and gender should also be carefully considered due to the difference in their restoration needs and restorative perceptions. The theoretical and practice-related contributions of this study can be further tested, adapted or extended in different contexts and among different children’s groups. The mechanisms undergoing the restorative benefits for children also need further research to better understand how diverse soundscapes offer the restorative experience to different children groups.

## Data availability statement

The original contributions presented in this study are included in the article/supplementary material, further inquiries can be directed to the corresponding author.

## Ethics statement

This study involving human participants were reviewed and approved by the Academic Committee of the School of Architecture, Tianjin University and Academic Committee of College of Architecture and Urban Planning, Qingdao University of Technology. Written informed consent to participate in this study was provided by the participants’ legal guardian/next of kin.

## Author contributions

SS: research idea and study design, data collection, data analysis, and manuscript writing.
